# Leaf Phenological Characters of Main Tree Species in Urban Forest of Shenyang

**DOI:** 10.1371/journal.pone.0099277

**Published:** 2014-06-25

**Authors:** Sheng Xu, Wenduo Xu, Wei Chen, Xingyuan He, Yanqing Huang, Hua Wen

**Affiliations:** State Key Laboratory of Forest and Soil Ecology, Institute of Applied Ecology, Chinese Academy of Sciences, Shenyang, China; University of Kent, United Kingdom

## Abstract

**Background:**

Plant leaves, as the main photosynthetic organs and the high energy converters among primary producers in terrestrial ecosystems, have attracted significant research attention. Leaf lifespan is an adaptive characteristic formed by plants to obtain the maximum carbon in the long-term adaption process. It determines important functional and structural characteristics exhibited in the environmental adaptation of plants. However, the leaf lifespan and leaf characteristics of urban forests were not studied up to now.

**Methods:**

By using statistic, linear regression methods and correlation analysis, leaf phenological characters of main tree species in urban forest of Shenyang were observed for five years to obtain the leafing phenology (including leafing start time, end time, and duration), defoliating phenology (including defoliation start time, end time, and duration), and the leaf lifespan of the main tree species. Moreover, the relationships between temperature and leafing phenology, defoliating phenology, and leaf lifespan were analyzed.

**Findings:**

The timing of leafing differed greatly among species. The early leafing species would have relatively early end of leafing; the longer it took to the end of leafing would have a later time of completed leafing. The timing of defoliation among different species varied significantly, the early defoliation species would have relatively longer duration of defoliation. If the mean temperature rise for 1°C in spring, the time of leafing would experience 5 days earlier in spring. If the mean temperature decline for 1°C, the time of defoliation would experience 3 days delay in autumn.

**Interpretation:**

There is significant correlation between leaf longevity and the time of leafing and defoliation. According to correlation analysis and regression analysis, there is significant correlation between temperature and leafing and defoliation phenology. Early leafing species would have a longer life span and consequently have advantage on carbon accumulation compared with later defoliation species.

## Introduction

Plant leaves, as the main photosynthetic organs and the high energy converters among primary producers in terrestrial ecosystems, have attracted significant research attention. This is due to their growth and characteristics that are able to affect basic behaviours and functions of plants directly. The leaves of the urban forest tree species, in particular, show special ecological functions and aesthetic value [1]. Previous studies on leaves mainly concentrated on their external morphology, internal structure, differentiation, pigment content, leaf position, phyllotaxis, and age which are related to photosynthetic physiology [2]–[6]. In recent years, research concerning the ecological functions of canopy and leaf has been of increasing importance. The optimal leaf lifespan model [7] and the comprehensive ecological mechanism simulation model of leaf lifespan at large-scale were proposed to show those climate-induced vegetation changes [8]–[9]. The structural and ecological characteristics of plant leaves, such as: leafing time, defoliating time, and leaf lifespan determined by both (belonging to leaf phenological parameters) can all reflect the temporal and spatial resource utilisation strategies of a plant [10]. Therefore, research on plant leaf morphology at different scales, and its ecology concerning leaf lifespan and related leaf characteristics are of ecological and evolutionary significance [11].

Leaf lifespan is an adaptive characteristic formed by plants to obtain the maximum carbon in the long-term adaption process. It determines important functional and structural characteristics exhibited in the environmental adaptation of plants [12]. The importance of leaf lifespan prolongation to plants' ecological adaptation can be analysed from the perspective of carbon accumulation. Zhu *et al*. [13] studied the leaf phenology of woody plants in a deciduous oak forest in Nanjing, China. They found that the plants adopted three ways to achieve leaf lifespan prolongation, including advancing leafing, delaying defoliating, and a combination thereof. Different species took different ways and generated various leafing and defoliating phonologies. The resource utilising strategies of plants were thereby reflected. For urban forests, due to the heterogeneity of site conditions and the inter-configuration complexity of species, there are different ways for different tree species in the carbon acquisition. Typical urban forests contain three layers, including: an arbour layer, a shrub layer, and a herb layer. Before canopy closure of arbour plants, shrubs and herbaceous plants fully absorb light resources for photosynthesis and complete their life histories [14]. Leaf lifespan prolongation is one of the major mechanisms of the successful invasion of alien species. McDowell [15] found that the leaf lifespan of *Rubus* invasive species were significantly longer than the native congeners. Many successful invasive species showed earlier leafing times and later defoliating times than native species. Furthermore, the leaves of some species were positive in spring and then developed into shade leaves after canopy closure. In addition, some herbivore-infringed plant species often delayed their defoliating time to increase leaf lifespan for more carbon acquisition [16]. Sun *et al*. [17] compiled statistics about the leaf groups of *Quercus liaotungensis* in the Donglingshan Mountains in China and compared the individuals of *Q. liaotungensis*. They pointed out that those individuals leafing early, defoliated later. Wang *et al*. [18] suggested that, possibly due to different light environments, the lifespans of small trees and shrubs exceed those of arbour species in the evergreen broad-leaved forest in Tiantong, Zhejiang, China. Although the undergrowth species in temperate broad-leaved forests leaf earliest and have the longest lifespan, the leafing time and leaf lifespans of different undergrowth species varies greatly. Some undergrowth species even display later leafing times and shorter lifespans than those of canopy species [13]. In 2004, Wright *et al*. [19] analysed the leaves of 2,548 plants species of various vegetation types distributed in arctic tundra, tropical rain forest, grassland, and desert: they covered 175 sampling points world-wide. For the first time, a universal relationship between plant leaf characteristic factors was explained on a global scale. However, the leaf lifespan and leaf characteristics of urban forests were not studied up to now. In recent years, we have carried out many studies related to urban forest in Shenyang [20]–[24], the largest forest urban in the northeast of China. At present, approximately 19 million trees have been planted in the city [25].

In this study, the trees in the urban forest in Shenyang Arboretum, Chinese Academy of Sciences, were observed for five years to obtain the leafing phenology (including leafing start time, end time, and duration), defoliating phenology (including defoliation start time, end time, and duration), and the leaf lifespan of the main tree species. Moreover, the relationships between temperature and leaf phenology, and leaf lifespan were analysed. The results obtained provided a scientific basis for Shenyang's urban forest construction and tree species selection.

## Materials and Methods

### Basic condition of the research area

The research was developed in the Shenyang Arboretum, The Chinese Academy of Sciences (41° 46′ N, 123° 26′ E), which is a densely populated commercial and cultural centre of Shenyang. It covers an area of about 5 ha and shows a warm temperate sub-humid monsoon continental climate, with four distinct seasons over the same period. Its annual average temperature is 7.4°C. The average temperatures in January and July are −12.6°C and 27.5°C respectively. The maximum temperature is 38.3°C and the minimum temperature is −30.5°C. This area shows urban forest microclimate characteristics [20]. Shenyang belongs to the North China flora area. However, it also lies in the intersection zone of Changbai flora and Mongolia flora. Thus the plant species are relatively rich. In Shenyang Arboretum, arbour and shrub species counts exceed 540 and at present, Shenyang Arboretum has developed into a typical urban natural forest with multiple layers and generations [14], [26]. Its forest community contains three obvious layers (arbour layer, shrub layer, and herb layer) or two layers (arbour layer and shrub layer). The arbour layer includes two sub-layers. The first sub-layer is about 12 to 22 m high. The tree species are comprise: *Phellodondron amurense*, *Robinia pseudoacacia*, *Quercus liaotungensis*, *Quercus mongolica*, *Fraxinus mandshurica*, accounting for 30% of the total trees in the arbour layer. The second sub-layer is 4 to 12 m high. The main species include: *Celtis bungeana*, *Prunus padus*, accounting for approximately 70% of the total trees in the arbour layer. The canopy density of the arbour layer is between 0.4 and 0.8. The trees having a diameter at breast height (DBH) of 4 to 32 cm are most frequent, making up 87.1% of the total, while those exceeding a DBH of 32 cm are rare, only accounting for 12.9% of the total. The main tree species in shrub layer include: *Lonicera maackii*, *Syringa velutina*, *Grewia parviflora*, *Lonicera praefloren*, *Evonymus alata*. The herb layer is mainly comprised: *Chelidonium dauricum*, *Viola yedoensis*, *Taraxacum ohwianum*, *Draba nemorosa*, *Polygonatum sibiricum*. The soil in the research area is a brown earth and is an alluvial meadow soil developed from the Hun River's deposits. The terrain is flat and free of the influence of underground water. Years of farming and 50 years of afforestation endow the forest with deep humus and fertile soil. During this experiment (2005–2009), the pH value of the soil of 0 to 40 cm deep is 6.3. The quick-acting nitrogen, quick-acting phosphorus, quick-acting potassium, and organic matter contents are: 147 mg/kg, 44.9 mg/kg, 294 mg/kg, and 2.54 mg/kg respectively.

### Research methods

In reference to “Chinese Phenological Observation Method” [27] and the growth characteristics of tree leaves, leaf phonologies were recorded by the same trained observers using: visual inspection, telescopes, and other special tools. Standard samples were selected and observed once a day on their south side using a randomised branch sampling method [28]. Leaf phenology observation lasted for five years from 2005 to 2009. Thirty-eight common tree species bearing Shengyang forest zonality were chosen for phenological analysis (Table 1).

**Table 1:** Leaf phonological characters of main tree species in urban forest of Shenyang (2005–2009 year).

Species	Beginning time of leaf emergence^a^	Ending time of leaf emergence	Duration of leaf emergence (d)	Beginning time of leaf abscission	Ending time of leaf abscission	Duration of leaf abscission (d)	Timing of leaf emergence	Timing of leaf abscission	Leaf life span (d)
*Ginkgo biloba*	43±9	68±8	21±7	209±4	225±2	16±3	45±8	218±5	173±3
*Phellodondron amurense*	49±5	78±4	29±4	223±5	237±5	14±1	51±5	231±4	180±5
*Fraxinus mandshurica*	54±3	75±3	21±2	210±2	226±3	16±5	56±3	214±15	158±14
*Fraxinus rhynchophylla*	49±5	71±±5	22±4	233±7	253±6	20±8	52±6	240±5	188±3
*Celtis bungeana*	55±10	77±10	22±4	253±3	261±2	8±2	57±3	256±2	199±15
*Juglans mandshurica*	46±4	71±4	25±6	220±10	232±12	12±3	48±7	226±11	17±14
*Quercus liaotungensis*	52±4	72±9	20±1	254±4	266±4	12±1	53±9	260±6	207±1
*Ailanthus altissima*	58±5	79±5	21±1	246±7	255±7	9±2	60±4	250±7	190±9
*Robinia pseudoacacia*	41±8	59±8	18±5	251±6	256±6	5±2	42±7	254±4	212±11
*Prunus padus*	21±11	51±10	30±7	217±1	228±2	11±1	23±11	224±1	201±10
*Ulmus pumila*	30±8	51±9	21±6	253±3	262±5	9±4	34±6	257±3	223±9
*Syringa amurensis*	37±8	66±6	29±6	232±3	249±5	17±7	40±5	238±4	198±7
*Xanthoceras sorbifolia*	47±1	74±4	27±3	239±4	251±3	12±5	50±1	245±4	195±4
*Sorbus alnifolia*	48±4	73±6	25±6	221±2	240±3	19±10	50±4	232±5	182±8
*Tilia mandshurica*	50±11	72±3	22±4	219±4	234±4	15±3	52±5	229±4	177±2
*Syringa wolfi*	47±10	59±5	12±3	250±5	260±2	10±2	38±7	255±2	217±4
*Cotoneaster multiflorus*	39±9	61±9	22±5	233±6	252±10	19±5	40±9	239±5	199±7
C. *melanocarpus*	32±9	60±9	28±3	232±3	246±6	14±4	35±8	239±5	204±3
*Lonicera maackii*	33±4	51±6	18±4	227±1	239±2	12±2	35±4	232±3	197±7
*L. praeflorens*	23±10	44±10	21±3	208±4	223±1	15±5	15±5	214±6	199±9
*Weigela florida*	52±5	72±2	20±3	229±13	250±10	21±9	54±4	250±10	196±10
*Evonymus alata*	51±5	66±4	15±2	237±4	251±3	14±2	53±5	244±4	191±7
*Syringa velutina*	33±7	54±6	21±2	250±5	263±5	13±7	35±7	256±2	221±9
*Viburnum sargenti*	38±9	59±9	21±2	243±2	256±3	13±3	40±10	251±2	211±9
*Grewia parviflora*	22±8	47±7	25±10	230±11	246±9	16±3	24±7	233±6	209±7
*Quercus mongolica*	51±6	78±9	27±9	241±4	251±2	18±4	54±7	253±5	199±7
*Cornus alba*	46±6	66±8	20±4	240±5	253±8	13±3	49±5	249±8	200±8
*Alnus tinctoria*	48±9	68±7	20±8	218±11	235±5	17±3	49±8	227±8	178±8
*Magnolia parviflora*	47±8	69±9	22±2	203±6	217±7	14±5	50±9	209±4	159±6
*Salix matsudana*	35±9	55±11	20±1	236±11	254±6	18±4	37±9	247±6	210±3
*Spiraca trilodata*	46±5	68±7	22±4	241±4	255±5	14±4	48±5	249±3	201±3
*Acer ginnala*	49±6	71±4	22±5	203±3	212±3	9±1	51±8	206±3	155±8
*A. pseudo-sieboldianum*	44±6	71±5	27±3	254±3	264±4	10±3	46±3	258±2	212±4
*A. triforum*	51±9	73±7	22±2	206±3	221±6	15±2	53±8	215±6	162±4
*Koelreuteria paniculata*	43±9	62±8	19±3	202±4	211±3	9±2	45±9	206±2	161±3
*Crataegus pinnatifida*	32±8	55±7	23±2	223±3	237±4	14±4	34±7	230±3	196±3
*Populus alba*	53±3	73±5	20±4	206±2	227±2	21±3	55±3	214±1	159±1
*Berberis amurensis*	38±8	62±11	24±6	232±4	252±5	20±5	42±8	245±5	203±8

a The timing for each leaf phenology is indicated by the days since March 1 every year from 2005 to 2009.

### Data processing

Firstly, leaf phenology was defined and related parameters were determined. Next, the leaf counts obtained at different times were standardised by referring to the leaf numbers in leafing and defoliating phases as the maximum values [13]. The leafing start time was set as the time that the average leaf count of a tree species reaches 10% of its maximum. Leafing end time is regarded as the time in which the average leaf count of a tree species reaches 90% of its maximum. Leafing duration referred to the time between leafing start and end time. Similarly, the start time, end time, and duration of defoliation were also set with 10% and 90% as critical values. Leaf lifespan is determined as the time between 50% leafing and 50% defoliating. The leafing time and defoliating time are defined as the time points with 50% leafing and 50% defoliating respectively. Finally, the means and standard deviations of the leafing phenology, defoliating phenology, and leaf lifespan in the five years were calculated (Table 1).

Meteorological data were collected from China's Meteorological Data Sharing Service System. The meteorological data in corresponding phenological phases were selected for this tree phenology study. Shenyang experiences spring, summer, autumn, and winter each year: these correspond to: March to May, June to August, September to November, and December to January respectively. Coldness index (CI) was calculated by the Kira heat index formula: CI =  (5 - *t*), where, *t* is the average monthly temperature over the year; CI is negative [29]–[30]. In this study, *t* is transformed into a ten-day average temperature (°C ten-day) [31]–[32]. The data were all statistically analysed and processed using MS-Excel and DPS (data process system).

## Results

### Leafing phenology

Statistics on the phenology observation data of the main tree species in Shengyang's urban forest [33] suggest that 73% of tree species begin to leaf at about the 15th day after sprouting (in the first ten days to the second ten days in April). However, different tree species differ greatly in leafing start time (Table 1). *G. parviflora*, *P. padus*, and *L. praeflorens* are the earliest leafing ones. They begin to leaf in the second ten days of March, which is 31 to 37 days earlier than the latest leafing ones, including *A. altissima*, *C. bungeana*, and *F.mandshurica*. Generally, the late leafing tree species leaf after blossoming, while the early leafing ones leaf before blossoming, or blossom and leaf simultaneously. Seventy-one percent of the tree species end their leafing in the first ten days to the second ten days of May. However, different tree species varies greatly in leafing end time. For example, to the second ten days in April, *G. parviflora*, *P. padus*, and *L. praeflorens* have finished their leafing, while many tree species had not yet begun to leaf. The latest leafing species, including *A. altissima*, *C. bungeana*, *P. amurense*, and *Q. mongolica* end their leafing processes by the last ten days in May, which is 26 to 35 days later than the earliest ones. The leafing process of the main tree species in this urban forest is concentrated. The leafing duration of 81% of the tree species is 18 to 25 days, and the leafing duration is 22 days on average.

**Figure 1:**
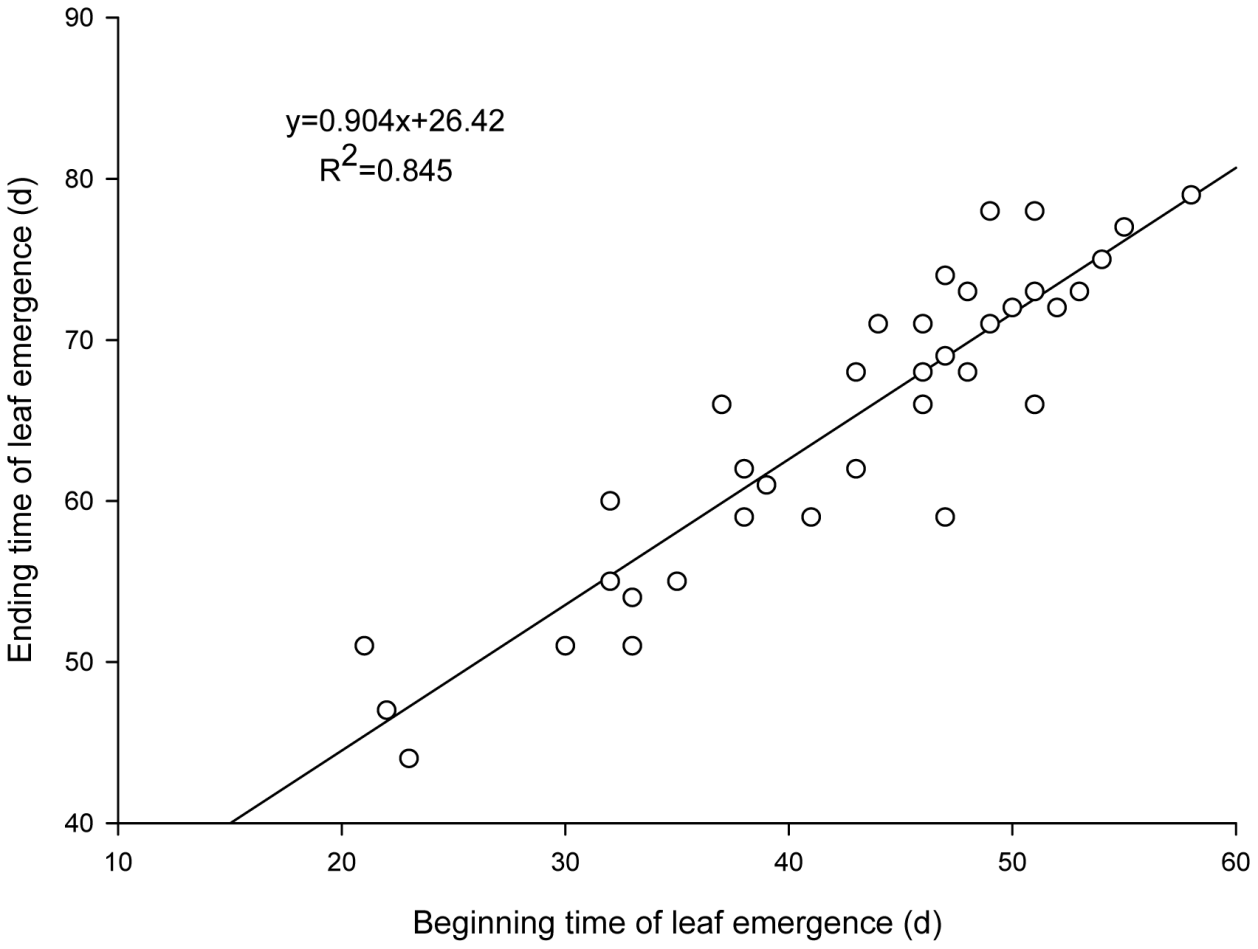
The relationships between beginning and ending time of leaf emergence.

Analysis of the leafing phenology of the main tree species in Shenyang's urban forest (Table 2, Figure 1) shows that leafing start time is positively correlated with leafing end time (*p*<0.01). That is to say, the earlier the leafing start time, the earlier the leafing end time; the longer the leafing duration, the later the leafing end time. However, leafing start time is independent of leafing duration. Thus it is proved to some extent that the early leafing tree species do not reduce their leafing speed and complete the leafing process earlier than those late leafing ones.

**Table 2:** Correlation analysis of leaf phenological characters of main tree species in urban forest of Shenyang.

	Beginning time of leaf emergence^a^	Ending time of leaf emergence	Duration of leaf emergence (d)	Beginning time of leaf abscission	Ending time of leaf abscission	Duration of leaf abscission (d)	Timing of leaf emergence	Timing of leaf abscission
Leaf life span(d)	−0.499**	−0.502**	−0.022	0.707**	0.722**	−0.239	−0.544**	0.737**
Timing of leaf abscission	0.108	0.111	−0.086	0.817**	0.970**	−0.110	0.103	
Timing of leaf emergence	0.991**	0.984**	−0.124	−0.067	0.090	0.084		
Duration of leaf abscission	0.090	0.115	0.103	−0.174	0.043			
Ending time of leaf abscission	0.096	0.101	−0.115	0.813**				
Beginning time of leaf abscission	−0.066	−0.038	−0.039					
Duration of leaf emrgence	−0.250	0.121						
Ending time of leaf emergence	0.975**							

a The timing for each leaf phenology is indicated by the days since March 1 every year from 2005 to 2009.

**p<0.01.

### Defoliating phenology

The main tree species in Shenyang's urban forest, accounting for about 25% of all tree species, begin defoliating in late September. These tree species are mainly sub-tropical or temperate plants, such as *M. parviflora*, *K. panicullata*, *A. ginnala*, *P. alba*, *G. biloba*. The latest defoliating time is observed in the first ten days of November. Related species are mainly native temperate species, including *Q. liaotungensis*, *U. pumila*, *A. pseudo-sieboldianum*, *C. bungeana*. Due to species differences, early defoliating time is about 44 to 52 days earlier than for late species (Table 1). However, the defoliating start time of most tree species is in October, accounting for about 58% of the total. In general, the defoliating end time of tree species in Shenyang's urban forest are concentrated: 87% of tree species end their defoliation between the second ten days of October and the first ten days of November. The defoliating durations of the tree species show little differences. The defoliating duration of about 52% of tree species is between 12 and 16 days. The average defoliating duration is 14 days.

**Figure 2:**
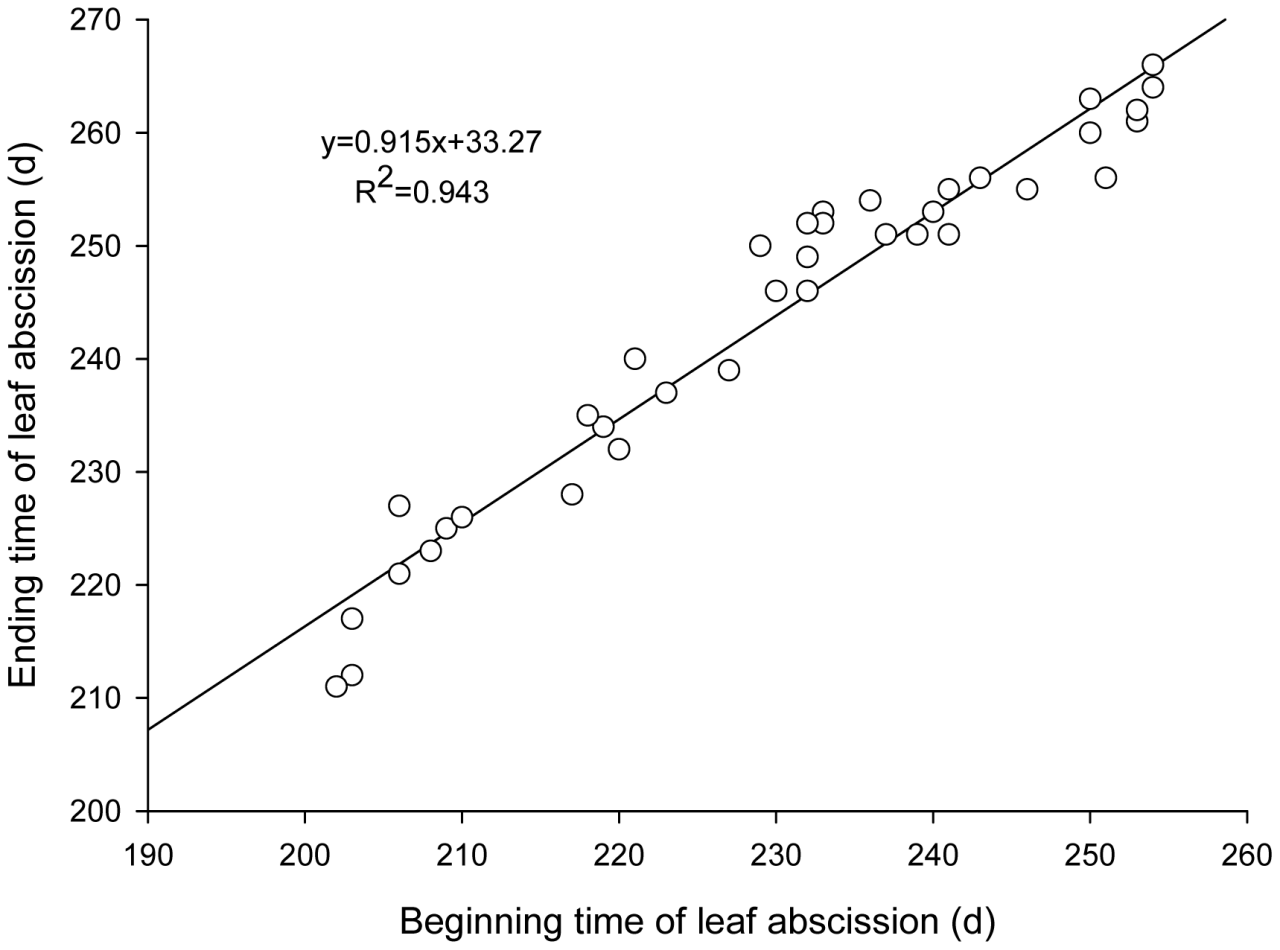
The relationships between beginning and ending time of leaf abscission.

Analysis of the defoliating phenology of tree species (Table 2, Figure 2) implies that the defoliating start time shows a significant positive correlation with defoliating end time and defoliating time (*p*<0.01). The earlier the defoliating start time, the earlier the defoliating end time. However, defoliating start time has nothing to do with defoliating duration. It is suggested that, to some extent, early defoliating tree species quickly reach a defoliating peak and defoliate quickly: the difference in defoliating end time is thus decreased.

### The relationship between leaf lifespan and leaf phenology

The main species in Shenyang's urban forest show an average leaf lifespan of 192 days. The leaf lifespans of 14 species exceed 200 days (Table 1), accounting for 37% of the total. *U. pumila* shows the longest leaf lifespan (223 days), while *A. ginnala* has the shortest at 155 days: a difference of 68 days. Analysis of the relationship between leaf lifespan and leaf phenology reveals that (Table 2), leaf lifespan is negatively correlated with leafing start time (*p*<0.05), leafing end time (*p*<0.01), and leafing time (*p*<0.01) at either a significant or very significant level. Moreover, leaf lifespan is positively correlated with defoliating start time (*p*<0.01), defoliating end time (*p*<0.01), and defoliating time (*p*<0.01) at a very significant level. That is to say, the earlier the leafing time and the later the defoliating time, the longer the leaf lifespan. However, leaf lifespan does not show significant correlation to leafing duration or defoliating duration. Therefore, in correlation analysis, we do not find significant correlation between the leafing time and defoliating time for these tree species (Table 2).

### The relationship between air temperature and leaf phenology

Plant leaf phenology is closely correlated with temperature. However, since plant phenological phases are strictly correlated, sequenced, and synchronized, plant leafing and defoliating occurrence has different temperature requirement, respectively [34]–[35]. For example, plants need to experience a series of events before leafing start, such as: sap flux, bud swelling, germination. As shown in Table 3, the leafing of the main species in Shenyang's urban forest is associated with the temperature of the last ten days of March and the first ten days of April, but almost no significant correlation with temperature in winter (November to February) is found. That is to say, the leafing of the tree species in this study is mainly influenced by the temperature of the spring before leafing instead of the winter temperature. However, leafing start time is positively related to the absolute value of CI (Table 3), which is closely to the distribution of vegetation. The larger the absolute value of CI, it means the later the leafing start time with higher energy and temperature requirements. According to the relationship of average ten-day temperature and leafing phenology, the linear regression equation of the leafing phenology of the main tree species in Shenyang's urban forest is: *y* = 5.827+0.493*x* (*R* = 0.901; *p*<0.01). It can thus be found that, with an increase in ten-day temperature of 1°C, leafing start time is advanced by 5 days. Nevertheless, the temperature requirements of the tree species are inconsistent due to their differing biological characteristics. The earliest leafing species, including *G. parviflora*, *P. padus*, *L. praeflorens*. leaf at an average ten-day temperature of 0.3°C in mid-March. While the latest leafing ones, such as *A. altissima*, *C. bungeana*, *F. mandshurica*. leaf at an average ten-day temperature of 11.2°C in late April. Up to mid-May, by when the ten-day temperature has reached 17.1°C, the tree species in Shenyang's urban forest have ended their leafing before warmer weather arrives.

**Table 3:** Relationships between phenology of leaf emergence and mean air temperature in ten days in urban forest of Shenyang (*n* = 38).

Month^a^	Sprouting	Timing of leaf emergence
11 I	0.017	0.103
11 II	−0.096	0.101
11 III	−0.213**	−0.103
12 I	−0.012	−0.037
12 II	0.272**	0.228**
12 III	0.045	0.143**
1 I	−0.248**	−1.020
1 II	−0.134*	−0.140*
1 III	−0.232**	−0.089
2 I	−0.254**	−0.200**
2 II	−0.401**	−0.327**
2 III	−0.346**	−0.361**
3 I	−0.083	−0.111*
3 II	−0.268**	−0.288**
3 III	−0.420**	−0.430**
4 I	−0.439**	−0.552**
4 II	−0.291**	−0.453**
4 III	−0.192**	−0.373**
5 I	−0.135*	−0.265**
5 II	−0.047	−0.163**
5 III	−0.119*	−0.123*
-*CI*	0.343**	0.193**

a I-The first ten days of a month, II-The middle ten days of a month, III-The last ten days of a month.

**p<0.01,

*p<0.05.

**Figure 3:**
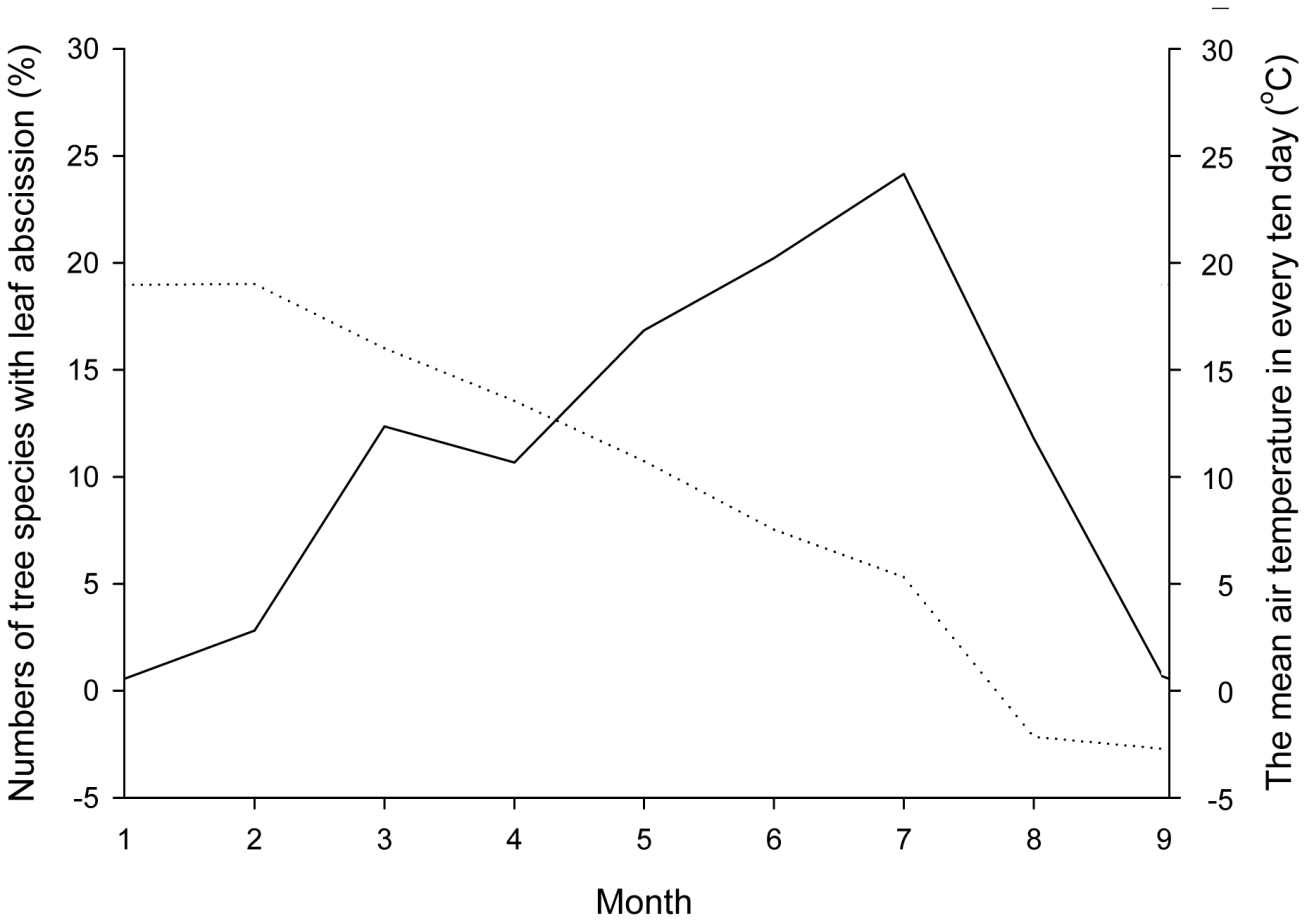
The relationships between leaf abscission phenology and mean air temperature in every ten days from September to November for main tree species in urban forest of Shenyang. The full line-numbers of tree species with leaf abscission (%), the dotted line-the mean air temperature in every ten day (°C).

The tree species here start to defoliate in late September. When the average ten-day temperature stays at 16.02°C, some tree species with high temperature requirements begin their defoliation, such as *K. panicullata*, *P. alba*, *G. biloba*. These species are the earliest to defoliate, accounting for about 12.36% of all defoliating tree species. Most of the tree species begin defoliation in October when the average ten-day temperature is between 7.64°C to 13.56°C. These species account for approximately 48% of the deciduous tree species. When the average ten-day temperature drops to 5.32°C in early November, 88% of the tree species enter their defoliation phase. Defoliation basically terminates at an average ten-day temperature of −2.16°C in mid-November (Figure 3). In accordance with the relationship of the defoliating phenology to average ten-day temperature, an equation is obtained: *y* = 71.706−0.266*x*; (*R* = 0.898; *p*<0.01). It can be found that, with the average ten-day temperature decreasing by 1°C, the defoliation of the tree species studied is advanced by 3 days Moreover, when compared with leafing, defoliation is more temperature-sensitive.

## Discussion

Leafing and defoliating phenologies experienced two eco-physiological processes of the periodical changes in plant leaf morphology. In these processes, plants capture carbon through leaf photosynthesis to provide for their growth, reproduction, and species maintenance [36]. Leaf lifespan, which is determined by leaf phenology, is a comprehensive index reflecting plant behaviour and function. It is considered as an adaptive strategy of plants developed in the long-term adaptation process aimed at obtaining the maximum photosynthetic yield and maintaining efficient nutrient utilisation [37]. The present study reveals that the leaf lifespan of the main tree species in Shenyang's urban forest differs due to the large differences in leafing and defoliating phenology. Leaf lifespan is significantly, and negatively, correlated with leafing start time, leafing end time, and leafing time, but is significantly, and positively, correlated with defoliating start time, defoliating end time, and defoliating time. However, leafing time and defoliating time did not show any obvious correlation. The results above suggest that plants can increase their leaf lifespan and carbon acquisition using two approaches: advancing leafing and delaying defoliating. Advancing leafing is of great ecological significances for the carbon acquisition of plants in a forest community and has often been researched. Seiwa [38] found that *A. mono* seedlings in a temperate deciduous broad-leaved forest have initiated their leafing two months before forest canopy closure. The carbon acquired in this time period was 61% to 79% of that accumulated in the whole year. Xu *et al.* [39] indicated that undergrowth invasive species *Berberis thunbergii* began to leaf two weeks before canopy closure, which is one month earlier than the native symbiotic species, *Vaccinium corymbosum*. In such a time period, *B. thunbergii* conducted photosynthesis using the early-spring sunlight in the forest. The carbon captured accounted for 36% of that accumulated in the whole growth cycle. Meanwhile, since the defoliating time of *B. thunbergii* was close to that of the native species, the advancing of leafing and long leaf lifespan of *B. thunbergii* increased its annual carbon accumulation and thus promoted the invasion. Kuppers [40] stated that the successful invasion and undergrowth settlement of *Ribes uvacrispa* were attributable to the strong competitiveness provided by early leafing instead of a high photosynthetic rate. This was because the early leafing of *R. uvacrispa* extended its leaf lifespan and advanced its carbon accumulation.

Plant leafing and defoliating processes are affected by genetic factors, environments, and biological factors such as insects, fungi. The defoliating phenology of the main species in an urban forest also determines the leaf lifespan. The later the defoliation, the longer the leaf lifespan. However, although leaf phenology is significantly correlated with leaf lifespan, defoliating phenology presents different carbon acquisition implications when compared with leafing phenology. Generally, leafing phenology means the unfolding of leaves, an increasing leaf area, an increasing leaf thickness, and finally an increasing net photosynthesis rate. Plant photosynthesis reaches a peak with the complete unfolding of leaves and then reduces with age [41]. At defoliation start time, carbon acquisition has dropped to practically zero. Measurement of the photosynthetic rates of the tree species in different phenological phases implies that the photosynthetic rate of leafing phenology is higher than that during defoliation. The average photosynthetic rate is 3.75 µmol•m^−2^•S^−1^ at the beginning of leafing, 7.93 µmol•m^−2^•S^−1^ at the end of leafing, 2.34 µmol•m^−2^ •S^−1^ at the beginning of defoliation, and almost zero at the end of defoliation. The results fully explained why the carbon accumulated during leafing is higher than that during defoliation. Meanwhile, it is also found that some early leafing native trees also have higher photosynthetic rates. For example, the maximum instantaneous photosynthetic rate of *S. matsudana* exceeds 17.31 µmol•m^−2^•S^−1^, and that of *U. pumila* is 22.43 µmol•m^−2^•S^−1^. By contrast, some north-moving tree species of North China's flora zone leaf late and show low photosynthetic rates. For instance, the maximum instantaneous photosynthetic rates of *G. biloba*, *S. oblate*, and *W. florida* are only 9.00 µmol•m^−2^•S^−1^, 8.13 µmol•m^−2^•S^−1^, and 6.59 µmol•m^−2^•S^−1^, respectively. These results show that the urban tree species with different phenological phases have different abilities of carbon consequence. Therefore, leaf lifespan and leaf phenology may play an important role in tree species selection for urban forest construction and sustainable development of urban forest under global climate change.

Plant phenological phases are closely associated with temperature. The starting time of each phenological phase in the plant's growth process is significantly correlated with the temperature before the phenological phase. Generally speaking, plant phenological phases are strictly correlated, sequenced, and synchronized. The later phonological phase is based on the former [32]. Therefore, the leafing time of tree species is determined by phenological phases such as: snap flux, bud swelling, and bud cracking. The leaf-expansion phase follows the bud germination phase. Before bud germination, a bud dormancy phase need a critical low temperature stage (a chilling process) to be completed. Zhu *et al*. [42] investigated the main meteorological factors that affect the phonological phases of the tree species in natural forests in Northeast China using principal component analysis (PCA). They considered that total cumulative sunshine hours and average temperature were two dominant factors inducing tree germination. Xie *et al*. pointed out that [43] average temperature was the main meteorological factor trigging tree germination. Xu *et al*. [1] studied the leaf growth relationships for the main tree species in Shenyang's urban forest. They found that the staggering of high and low temperature periods was the main meteorological factor giving rise to tree germination, and the low temperature period before germination was of great significance as a factor therein. Moreover, the critical low temperature of the main tree species in Shenyang's urban forest is observed between late January and late February (Table 3). It is a proper low temperature stage, but not the minimum temperature in Shenyang (this occurs mid-January). In the present study, the CI in the Kira heat indices was introduced to this phenology study to explore how the temperature before a plant phenology phase influenced its germination and leafing time, and how plant phenology interacted with each other. The results showed that CI was of ecological significance for studies on spring phenology. Xu *et al*. [44] revealed that plant bud germination was negatively correlated with winter temperature. Germination was advanced with the increase of winter temperature. Besides, the reduction of the minimum winter temperature was favourable to bud dormancy breaking and thus advanced the germination phase. Hannien [45] also pointed out that the germination of the plants in spring was mainly affected by the critical low temperature in winter, and this low temperature was conductive to breaking the bud dormancy. However, Zhang [46] considered that the winter temperature was unfavourable to bud dormancy and thus postponed the blossoming and leafing time. At a plant physiology level, leaf phenology is initiated by different factors. It is generally believed that leafing is closely connected with temperature and dominated by accumulative temperature particularly, while defoliating phenology is influenced by low temperature and photo-period. It is possible that defoliation is caused by the abscisic acid (ABA) in chloroplasts generated by the interaction of low temperatures and photo-period. However, some eco-physiological mechanism problems, such as how ABA is transferred by low temperature and photo-period signals, and how ABA influences defoliation, remain to be studied.

## Conclusions

In the present study, we find that there are significant correlations between leaf longevity and leaf phenology, and between temperature and leaf phenology. Early leafing species would have a longer life span and consequently have advantage on carbon accumulation compared with later defoliation species. The timing of leafing differed greatly among tree species. The early leafing species would have relatively early end of leafing. The longer it took to the end of leafing would have a later time of completed leafing. The timing of defoliation among different species varied significantly, the early defoliation species would have relatively longer duration of defoliation. We also find that if the mean temperature rise for 1°C in spring, the time of leafing would experience 5 days earlier in spring. If the mean temperature decline for 1°C, the time of defoliation would experience 3 days delay in autumn.
